# Co-designing the integration of voice-based conversational AI and web augmentation to amplify web inclusivity

**DOI:** 10.1038/s41598-024-66725-3

**Published:** 2024-07-13

**Authors:** Emanuele Pucci, Ludovica Piro, Isabella Possaghi, Davide Mulfari, Maristella Matera

**Affiliations:** 1 Politecnico di Milano, Milan, Italy; 2 Università degli Studi di Messina, Messina, Italy; 3 Norwegian University of Science and Technology, Trondheim, Norway

**Keywords:** Inclusive design, Conversational AI, Automatic speech recognition, Web augmentation, Engineering, Energy science and technology

## Abstract

The Web has become an essential resource but is not yet accessible to everyone. Assistive technologies and innovative, intelligent frameworks, for example, those using conversational AI, help overcome some exclusions. However, some users still experience barriers. This paper shows how a human-centered approach can shed light on technology limitations and gaps. It reports on a three-step process (focus group, co-design, and preliminary validation) that we adopted to investigate how people with speech impairments, e.g., dysarthria, browse the Web and how barriers can be reduced. The methodology helped us identify challenges and create new solutions, i.e., patterns for Web browsing, by combining voice-based conversational AI, customized for impaired speech, with techniques for the visual augmentation of web pages. While current trends in AI research focus on more and more powerful large models, participants remarked how current conversational systems do not meet their needs, and how it is important to consider each one’s specificity for a technology to be called inclusive.

## Introduction

With the progressive emergence of conversational agents (CAs) and virtual assistants, voice user interfaces have increased their presence in our everyday activities^1^. Their natural-language paradigm simplifies the interaction with digital systems and offers benefits in several situations where users may take advantage of speech interaction, extending the inclusivity of technology to users living with permanent or situational disability^2,3^.

Over the years, several studies have shown the opportunity to adopt CAs for exploring the Web, focusing on aspects such as designing conversations for searching the Web^4^, generating automatically CAs out of single web page content^5^, or enabling end users to customize their CAs for extracting content from the Web^6^. This interest shows the feasibility and potential of adopting CAs for making the Web truly for everyone. However, as of now, CAs do not focus on website browsing, and the fruition of web pages is limited to a sequential content reading^7^. A recent work^8^ introduces a novel paradigm for conversational web browsing, *ConWeb*, that enables users to browse the Web through conversation. Browsing goals and intents for web content access can be expressed through a voice-based dialog with a CA, thanks to conversational patterns that define how users’ natural-language queries can guide web browsing. The same research shows the effectiveness of this conversational paradigm for people with visual impairments. However, in the presence of atypical speech patterns, voice interaction may result in a technological barrier.

Among speech impairments, dysarthria is a common neurological disorder that interferes with articulation, respiration, phonation, and resonance. Individuals’ speech intelligibility is negatively affected since it is characterized prosodically as having monoloudness, monopitch, impaired ranges of the fundamental frequency, rate, and vocal intensity^9^. Often, severe motor impairments, such as miscoordination or inaccuracy of articulatory movements, and limited sight co-exist. Individuals living with dysarthria would benefit from voice-based interaction. Still, speech-production irregularity compromises Automated Speech Recognition (ASR) by the largely adopted conversational AI models, which are trained on samples of typical speech.

To cope with such challenges, this paper proposes a multimodal interaction paradigm that extends previous work on Conversational AI for web browsing^3^ to support people with dysarthria when accessing the Web. The proposed approach combines: (i) a *conversational paradigm* defined on top of a model for speech recognition purposely trained for a vocabulary of impaired-speech words, and (ii) a *visual augmentation paradigm* that extends web pages with overlays of visual labels highlighting the vocal keys, belonging to the vocabulary of the recognized words, that can trigger browsing actions on the visited web page.

The new paradigm is the result of a human-centered process that, following a “research-through design” approach^10^, involved people with dysarthria to co-design and evaluate with them incremental prototypes that progressively highlighted relevant challenges and possible design solutions. The paper outlines the design dimensions and related patterns that characterize the new multimodal paradigm for web browsing. A relevant contribution is the design process in which a sample of people with dysarthria actively co-designed and validated technology for them. While the literature reports user-based studies to create dysarthric speech datasets and assess the accuracy of ASR, our work is one of the few attempts to conduct human-centered, participatory design processes. Our goal is to enhance browsing experiences for individuals with disabilities to make the Web inclusive. While current AI research focuses on powerful models and sophistication, it often overlooks the specific needs of individuals living with disabilities. Promoting formative evaluations during and after the development of such technologies can address this gap and help develop tailored AI systems to promote inclusivity and fairness. Starting with smaller-scale research helped us build such a rigorous formative process. Thanks to our methodology, we could focus on stringent requirements and, at the same time, discover unique innovative solutions that benefit everyone.

The paper is organized as follows: The section on “Related works” discusses relevant related approaches for the design of conversational agents for the Web and Web Augmentation. Section “Design process” illustrates the design activities that led to the identification of challenges for web browsing and design dimensions for multi-modal interaction. Section “Patterns for multimodal web browsing” describes the resulting design patterns, and “Implementation” explains their integration within a web platform enabling multi-modal web browsing. Section “User-based validation” reports on a preliminary validation of the identified solution and “Discussion” deepens the main implications for designing intelligent systems for inclusivity, and the limitations of the conducted design process and of the resulting implementation. Section “Conclusions” finally draws our conclusions and outlines relevant future work toward defining human-centered design methods for intelligent technology.

## Related works

Multimodal interaction has been studied since the beginning of this century to enable a more natural communication accommodating diverse users’ needs^11^. Concerning web access, multimodality has been investigated to ensure accessibility and inclusivity^12^. However, despite past attempts to propose new web standards and mark-up languages for multimodal interaction on the Web and to extend web browser capabilities to address accessiblity, there is still a lack of unified and at the same time flexible frameworks enabling web access through modalities and devices that best suits the users’ needs. Significant are the experiences of the voice (https://www.w3.org/Voice/) and multimodal (https://www.w3.org/2002/mmi/) W3C working groups that, however, have been dismissed in 2015. The same happened for another initiative brought forth by the browser Opera (https://dev.opera.com/blog/opera-accessibility-where-we-re-at/). However, current intelligent technologies can now contribute to the definition of robust solutions. By exploiting the latest advancements in the field of voice-based conversational AI, our research addresses this gap by combining *Web augmentation* with *voice interaction* to address voice and motor impairments of users living with dysarthria. Relevant works focusing on these two interaction channels are discussed in the following.

### Bringing conversational AI to the Web

Conversational agents (CAs) are pervading our everyday activities, as their natural-language paradigm simplifies the interaction with digital systems and offers benefits in varying situations where users may take advantage of voice-based interaction for accomplishing their tasks^13,14^. Often, CAs are integrated within websites to offer parallel channels to access the website content. However, such CAs do not support website browsing nor the direct fruition of page content^7^. Nonetheless, recent works are capitalizing on conversational technology, for example, to design voice-based CAs for searching the Web and activating the web browser functions through purposely-designed browser extensions^4^. Other works present methods for automatically generating CAs from website content thanks to custom HTML annotations^5^. Lastly, research has also leveraged end-user development approaches, such as programming by demonstration or visual annotation of the web page HTML, to enable end users to create their CAs for accessing a specific website^6,15,16^. While this interest shows the feasibility and potential of CAs for making the Web accessible to everyone, the proposed approaches present limitations regarding accessibility. First of all, these tools are not explicitly designed for inclusivity. Furthermore, the proposed end-user development approaches require manual intervention on each website, which can pose challenges or render them inaccessible for users with motor impairments.

*Conversational Web Browsing* (*ConWeb)* is a new paradigm that enables users to express their web browsing goals and access websites through dialog-based interactions with a CA, instead of operating graphical UIs using keyboards, mice, or screen readers^5^. Web browsing CAs are automatically generated without requiring their manual definition, neither by the designer nor by the final user^8^. User studies have shown this conversational paradigm is effective for people with visual impairments. *ConWeb* could, in principle, benefit users living with dysarthria, since the motor impairments that characterize this condition can take advantage of vocal interaction. However, CAs trained on typical speech patterns are inadequate for understanding dysarthric speech. As the following sections will show, adopting automatic speech recognition (ASR) models that are trained *ad-hoc* for speech impairments is essential to overcome these barriers. The conversational patterns must also be tailored to address an additional requirement: the limited vocabulary that even ad-hoc trained ASR technology can interpret. The solution illustrated in this paper proposes the coordination of the dialog system with a visual augmentation mechanism that, at each conversation state, delimits and visually highlights the page portion in which the few available commands allow the users to select inner elements.

### ASR for people with dysarthria

Depending on the severity of their neuromotor disabilities, people with dysarthria can rely on assistive technologies (ATs) addressing reduced motor skills^17^. Notable examples are on-screen keyboards, adaptive keyboards, adapted joysticks, head-trackers and eye-trackers^18^. Internet of Things (IoT) is recently being leveraged to automate daily tasks at home, some even leveraging Automatic Speech Recognition (ASR) for voice-commands recognition^19,20^. Voice interfaces could help when motor disability is severe. However, as of now, the effectiveness of ASR technology in the presence of atypical speech patterns is still quite limited. Within the related literature, studies demonstrated that the current speech recognition tools might work well for mild dysarthria^21^, but the performance degrades as the severity of the speech impairment grows up. As an example, the *TORGO* dysarthric speech dataset^22^ was adopted to study the accuracy of the impaired speech recognition as achieved by three speech recognition cloud platforms^23^: IBM Watson Speech-to-Text, Google Cloud Speech, and Microsoft Azure Bing Speech. The authors proved the limits of these platforms when dysarthric speech intelligibility is low (80–90% of word error rate). Primary factors influencing this reality include the scarcity of dysarthric speech datasets^24^.

Nonetheless, some works propose ASR specifically designed for users with dysarthric speech. *CanSpeak* is an interface to interact with software applications thanks to a set of keywords that can be associated with the available commands. The user’s input speech is mapped to keywords that the user or the caregiver can customize^25^. *ALADIN* proposes an adaptive ASR addressing home automation and entertainment^26^. Starting from a predefined data structure, the system learns from users how to recognize the commands according to the grammar and vocabulary they prefer the most; over time, it then becomes more and more accurate. The *VIVOCA* system^27^, instead, is a voice-input voice-output application that uses ASR to interpret the speech uttered by the users and render it in a synthesized voice.

In the context of the Italian language, Human-Computer Interaction (HCI) research for ASR applications is scarce. Furthermore, the available speech data sets are very limited. The *CapisciAMe* project is an initiative to investigate the adoption of supervised Machine Learning approaches in recognizing the Italian impaired speech^28^. To approach the typical challenges behind ASR, the project focuses on isolated word recognition tasks, and, due to the lack of Italian dysarthric corpora, a crucial addressed aspect regards the speech collection from individuals with dysarthria who are weak in voice production due to their physical disabilities^29^. At present, speech data acquisition is an ongoing activity and, in particular, with the collaboration of 170 native Italian speakers with speech disorders, the corpus consists of 54K single recordings split into 65 classes. Thanks to this corpus, deep neural network models have been trained to achieve an ASR system capable of classifying dysarthric utterances in one of the keywords within the ASR dictionary. Such an inference task has been recently deployed as a cloud service, with the final goal of empowering application scenarios involving end users with speech disabilities. Implementing the multimodal paradigm illustrated in this paper takes advantage of this service for recognizing vocal commands pronounced by people with dysarthria.

### Web augmentation

Web Augmentation (WA) allows users to customize their experience on the Web, offering extensions that add new content and functionalities to web pages. Web Object Ambient (WOA)^30^, for example, proposes an End-User Development (EUD) approach to personalize the experience in e-commerce websites. Server- and client-side components allow the users to annotate page elements to add product reviews or comments. The user-generated content is saved server-side and shown to other users looking up the annotated product. This approach shows the potential of Web Augmentation for personalizing web access; however, very few works address accessibility, and in particular the needs of people living with neuromotor impairments^31^.

Farfalla^32^ is a web browser plug-in that changes the page color scheme, text size, and text type based on the user’s preferences and adds a virtual keyboard. WAFRA^33^ is a client-side framework designed for information-dense websites, such as Wikipedia, that allows users to access web content through a voice interface. Thanks to a set of predefined operations, the framework reads aloud a piece of content and enlarges text size. To our knowledge, there is a lack of approaches addressing web accessibility for people living with neuromotor impairments^31^.

### Human-centered design of voice-based conversational AI for inclusivity

We believe our work can advance the state of the art in three directions:

- First, works evaluating ASR performance for people with dysarthria do not assess such systems with real users, yet use state-of-the-art datasets, e.g., Torgo^34–36^ or UA-Speech^37^. Working side-by-side with a group of participants with a speech disorder helped us focus on specificities that could not have arisen otherwise.
- Second, most of the datasets that can be used for testing ASR performance are English-language datasets^22^. To advance the inclusivity of technology, working with local languages is paramount. That is why our framework integrates the *CapisciAMe* ASR technology^38^.
- Third, while most of the works concentrate on the evaluation of technical performance a minimal number of studies focus on co-designing solutions and speech-controlled interfaces for people living with dysarthria^25,26^.

With our contribution, we hope to push forward the application of human-centered approaches to develop intelligent systems that are conceived *for* and *with users living with impairments*. In the case of dysarthria, this is very challenging due to (i) the difficulties in recruiting participants and (ii) the wide variety and the diversity of needs of each participant. These challenges, especially the low number of involved participants, are documented in almost any work dealing with human-centered design and evaluation studies^25,39,40^. Despite these difficulties, we believe this is a promising direction in which we can strive to address the specificity of each individual and make technology truly inclusive.

## Design process

The research described in this paper capitalizes on a conversational paradigm for web browsing previously defined for Blind and Visually Impaired (BVI) users^41^ and supported by the *ConWeb* platform^8^. The results achieved in this previous research showed the technological feasibility of browsing the Web through voice interaction.

To understand how to accustom this initial paradigm to the needs of people living with dysarthria,

we involved 5 participants living with this condition (self-identified as males), reached out through the association *TecnologicaMente InSuperAbili* (Technologically unsurpassed, in English, https://www.tecnologicamenteinsuperabili.org/). With them, we conducted a series of formative studies from September 2022 to January 2023, under the authorization of the research ethical committee of *Politecnico di Milano* (Opinion no.11/2021). Informed consent was obtained from all participants included in this study, in accordance with ethical guidelines and institutional requirements, ensuring their voluntary participation based on their understanding of the study’s objectives, procedures, required effort, and benefits.

**Figure 1:**
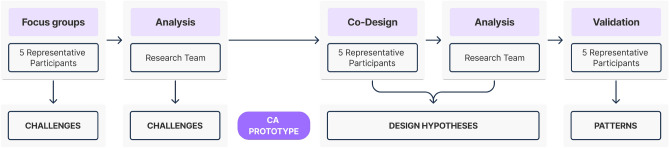
The human-centered process for the design of the multimodal browsing paradigm. It represents a scheme comprising the steps that guided our analysis, with the relative participants and output. We have the focus groups and its related analysis, the co-design and its related analysis, and the final validation of the defined multimodal browsing patterns.

As illustrated in Fig. 1, we followed a “research-through-design” approach^10^, with focus groups and co-design sessions in which the participants expressed their challenges in interacting with *ConWeb* and directly acted on prototypes to explain their needs, expressing desiderata in the form of alternative solutions. The insights gained in the different steps progressively guided the identification of a new paradigm that adapts the conversational web browsing patterns and extends them with web augmentation. Due to the participants’ specific needs, it was impossible to run sessions with many of them. To give space to each participant, the researchers then organized two focus groups and two co-design sessions, one with 2 participants and one with 3 participants.

### Focus group

In the middle of September 2022, we invited the members of the *TecnologicaMente InSuperAbili* group to participate in the focus group. We reached out to them thanks to the volunteers’ association *Informatici senza Frontiere*. 5 individuals aged between 24 and 39 years accepted, all self-identified as male. Table 1 shows how their impairments vary significantly.

**Table 1:** Participants’ data: age, impairment, and adopted assistive tools.

Participants	Impairments	Assistive Technology
P1 (age: 39)	Limited sight and motor capacity	Can use a virtual keyboard thanks to the mouse
P2 (age: 25)	Limited sight and motor capacity	Can use a virtual keyboard thanks to the mouse
P3 (age: 38)	Limited sight and motor capacity	Can use the computer keyboard and the virtual keyboard thanks to the mouse
P4 (age: 24)	Optimal sight and limited motor capacity	Can use a virtual keyboard thanks to the mouse
P5 (age: 37)	Optimal sight and limited motor capacity	Can use the computer keyboard with a keyguard

The Table reports the information about participants: their age, the severity of the impairments, the type of assistive technology they can use.

The two focus group sessions were conducted online using Cisco WebEx and lasted two hours and a half. Given possible difficulties in interpersonal communication, one parent for each participant and one volunteer of the *Informatici Senza Frontiere* organization attended the call. Two researchers moderated the session.

The focus groups started with every participant presenting himself. They all declared to be highly skilled in Information Technology (IT); two of them have got a Master degree in Computer Engineering. They all said to be familiar with CAs, e.g., Alexa and Google Assistant but, in line with the findings of previous studies addressing virtual assistants’ capabilities with impaired speech^39^, they expressed their frustration when interacting with them.

The researchers then introduced the participants to the core concepts of the *ConWeb* platform and conversational Web browsing and showed the paradigm at work on a few Wikipedia web pages about the solar system. The aim was to understand to which extent that paradigm could still be helpful, and identify the **main challenges** brought by the transposition of *ConWeb* to impairments not considered before. A video demonstrating the scenario used for this introduction is available at: https://tinyurl.com/demo-validation.

As shown in Table 1, one condition participants live with is the limited motor ability that makes it difficult to move a mouse (**P2**: *“It can be very challenging, if not impossible, to interact with an individual element on the page, especially the drop-down menus”*) or type on a keyboard (**P3**: *“Writing whole words on the keyboard is tiring, especially when you can’t reach all of the letters.”*). Voice user interfaces can help; however, the participants observed that actual CAs are not thought to be used once the users land on a web page (**P4**: *“Google Assistant is really easy to use, but unfortunately, it’s not conceived to tell me about web pages as well: it only goes so far as to search [on Google]”*). The *TecnologicaMente InSuperabili* team was very interested in voice interaction for web browsing. Still, at the same time, they recognized how their condition affects the capacity to speak clearly, with subsequent difficulty in using traditional CAs (**P1**: *“Alexa’s voice recognition fails with me: the words I speak are not recognized. Furthermore, its listening time is very short and I need time to tell entire sentences”*).

The researchers then discussed with the participants how they would access content on the Web (e.g., reading an article, moving through different pages), and what the main obstacles could be if navigating the websites by using *ConWeb*. While moderating these activities, the researchers individually took notes on significant participants’ behaviors and aloud comments. After transcribing their notes and post-experience considerations, they independently conducted an inductive thematic analysis. Then, they reduced a few variations on the emerging themes (10%), till reaching a complete agreement on the following challenges.

#### Speech impairment

For the participants, the first concern with conversational browsing was the difficulty of communicating their navigation intents, since current ASR technology is not trained to comprehend atypical speech. Our collaboration with the *TecnologicaMente InSuperabili* team was born to overcome this stumbling block. In fact, the group includes the creator of the CapisciAMe^38^, an ASR engine purposely trained to spot voice commands from users with dysarthria. The participants observed that even with CapisciAMe a persisting challenge would be the limited vocabulary CapisciAMe can understand, which amounts to the 10 digits plus a few dozen other single words.

#### Motor impairment

The participants’ motor impairments are different. One challenge was thus to analyze how each participant would browse the Web given the specific level of motor impairment. The researchers extensively analyzed with the participants the possible modalities with both physical and on-screen keyboards, for example, whether they would benefit from shortcuts to activate given functions.

#### Visual impairment

As for motor impairments, visual impairments can also differ in severity from one individual to another. In addition to providing the 3 visually-impaired participants with the *ConWeb* conversational paradigm, we wanted to understand whether the visual channel combined with the conversation could be helpful for the sighted participants. Our hypothesis, based on what we observed in previous studies with visually-impaired (not blind) users, was that the combination could improve orientation while browsing, which in the conversational-only paradigm had emerged as a complex aspect^8^. The 3 visually-impaired participants stated they could grasp what is written on a web page and that, if properly supported, would benefit from the written content and the visual layout organization to improve their navigation experience.

Table 2 summarizes the main dimensions investigated during the focus group and the main findings that informed the next phase. The primary outcome was that a *pure vocal interaction* would limit most participants’ experience. Thus, the researchers started elaborating on a paradigm offering vocal and visual interaction but with nuances in the two channels addressing the observed speech and motor impairments.

**Table 2:** Main dimensions investigated during the focus groups, together with the relative challenges for the extension of the framework.

Main dimensions	Challenges
Speech impairments	- Difficult voice interaction due to speech-to-text capabilities - Available speech-to-text systems only support a very limited number of words
Motor impairments	- Motor capabilities greatly vary among dysarthric people - The majority of the participants expressed the will to have a minimum interaction with the keyboard
Visual impairments	- Visual capabilities greatly vary among dysarthric people - Visual capabilities, on average, are more developed than BVI people - Participants wanted to leverage their visual capabilities to improve the interaction with the assistant

The table reports the main dimensions investigated during the focus groups on the left column (speech, motor, and visual impairments), together with the relative challenges for the framework extension in the second column.

### Co-designing a CA

The two co-design sessions were held with the same participants and researchers two weeks after the initial activities. The goal of the 2-hour online sessions was to gather insights on how to structure a conversation for web browsing, given the vocabulary of words that the CapisciAMe ASR could interpret. The participants were asked to design with the researchers a conversation about browsing a website of their choice. They chose their own organization website, which they claimed to be fully compliant with the accessibility guidelines, yet not accessible for them. As soon as the first ideas emerged, the researchers helped the participants implement the conversations with the rapid-prototyping tool *VoiceFlow*, available at https://www.voiceflow.com/. Participants could run the prototype and play with it. Supported by the researchers, they iteratively modified it to express and refine their ideas.

The co-design session material was organized and analyzed through a new inductive thematic analysis that revealed multiple insights, all revolving around two main aspects: *simplified voice navigation* and *mixed-interaction mode*.

**Simplified voice navigation**. Following the focus group discussion, the participants asked for a vocal interaction for web browsing using a limited set of commands. It was then necessary to identify ideas and strategies to browse content using the CapisciAMe vocabulary. For example, **P2** stated *“Numbering elements in the page would work; in this way, it is easy to choose the elements to explore!”*, followed by **P3** affirmation: *“Locating a paragraph that I want [to read] can be faster and more effective if I can pronounce a digit associated with the title of that paragraph.”* The participants outlined the list of words they would use. Then, the researchers, guided by the participants, adapted the *ConWeb* navigation patterns^8^ to the new needs. In particular, every browsing intent was linked to one item of the “word list” provided by the group. The mapping between browsing intents and the words was accurately weighted with participants. **P4** said: *“In my opinion, sticking to digits instead of words like “up” and “down” is far better; otherwise, selecting elements in complex [layout] pages would be extremely confusing”*, and **P3** stated:*“The use of “go on” when already reading a text is quite straightforward, we can avoid pronouncing digits and remain simpler in this situation!”*.

**Mixed-interaction mode**. The participants with a less severe visual impairment asked for a system leveraging their sight abilities. They did not want to passively receive the entire information through the speech channel. They wanted to be **visually supported** to match the website information with the information coming from the speech channel (**P4**:*“I’d like to figure out where page elements are located without the conversational agent listing everything for me”*, and **P1**: *“If I have an overview of choices by digits, it will be straightforward to navigate!”*). They also wanted to move easily within the page content, for example, to skip one paragraph. The visual channel could help them understand what is (or is not) attractive to them (**P1**:*“It would be interesting to make the digits visible on the screen, so if I want to stop reading [this paragraph], I can change topics quickly by pronouncing another number”*). The opportunity of using the keyboard also emerged, even if minimum and limited to a *one-key* click and only with a *predefined set of keys*.

Table 3 summarizes the findings of the co-design sessions for each of the challenges previously identified. The participants designed CAs that:

- Can understand their language and support them in the website navigation by leveraging ASR technology and a “*navigation-by-digit*” mechanism.
- *Superimpose graphical elements* (such as digits or strokes) to highlight page elements on which they can operate through pronounced or typed digits.
- Enable a* hybrid interaction*, in part through voice and in part through the keyboard, depending on situational needs.
- Let the user *customize the paradigm* on its preferences and needs.

**Table 3:** Design opportunities emerged during the co-design sessions for each identified challenge.

Challenges	Co-designed (user-defined) CA characteristics
- Difficult voice interaction due to speech-to-text capabilities - Available speech-to-text systems only support a very limited number of words	- Integration with CapisciAMe - New information architecture to let the user navigate the content “through digits”
- Motor capabilities greatly vary among dysarthric people - The majority of the participants expressed the will to have a minimum interaction with the keyboard	- Possibility to interact with the CA not only by voice, but also by typing shortcuts on the keyboard to improve the browsing experience
- Visual capabilities greatly vary among people with dysarthria - Visual capabilities on average more developed than visually-impaired people - Participants wanted to leverage their visual capabilities to improve the interaction with the assistant	- Mixed interaction mode - Highlight of visual elements while browsing - New onboarding process to adjust the CA capacities to the user’s needs

The table reports the main challenges identified during the focus groups on the left column and the co-designed CA characteristics on the right column.

## Patterns for multimodal web browsing

*Conweb* was built around conversational patterns covering typical web browsing aspects: orientation, link navigation, content location within pages, and content summarization^8^. Based on the insights gained through the co-design sessions with the *TecnologicaMente InSuperAbili* group, the researchers considered how to revise those patterns to achieve a new multimodal paradigm with a level of flexibility satisfying the different needs emerged during the formative activities. They separately performed a deductive thematic analysis, clustering codes from *in-vivo coding*^42^ into the themes already identified in the previous phases. They discussed together, to solve minor disagreements and some aspects clearly emerged from their analyses. In particular, while touching “in-the-small” features, such as personalizing the pitch speed and the layout of the graphical interfaces, the participants constantly expressed the need for different interaction modalities and the desire to choose the (combination of) modalities that would best suit their specific needs. **Multimodality**, intended as the (i) provision of multiple paradigms, with the opportunity to (ii) configure each paradigm and (iii) opt for paradigm combinations, was identified as a high-priority dimension, which could transform web browsing from a pure vocal to a *mixed experience*.

Then, within the general need for multiple modalities, the opportunity for a tight combination of vocal and visual browsing also emerged. The addition of visual labels suggesting the keys to be pronounced or typed to explore or activate specific page elements emerged as the most prominent solution. The gathered insights thus motivated the definition of the design patterns we describe in the following, organized according to the main themes that emerged from the formative activities.

**Limited-Vocabulary Conversation.** Users can interact with the original *ConWeb* platform in natural language without limitations on the used terms and expressions. This is the interaction modality for which the *ConWeb* platform was originally designed. The *Limited-Vocabulary Conversation* pattern expands the traditional approach thanks to the integration with the CapisciAMe service^38^. Together with the *TecnologicaMente InSuperAbili* group the researchers selected the most suitable and intuitive CapisciAMe words to be mapped on intents for web browsing. A list of the words and commands can be found at: https://tinyurl.com/Dysarthria-VoiceCommands.

**Figure 2:**
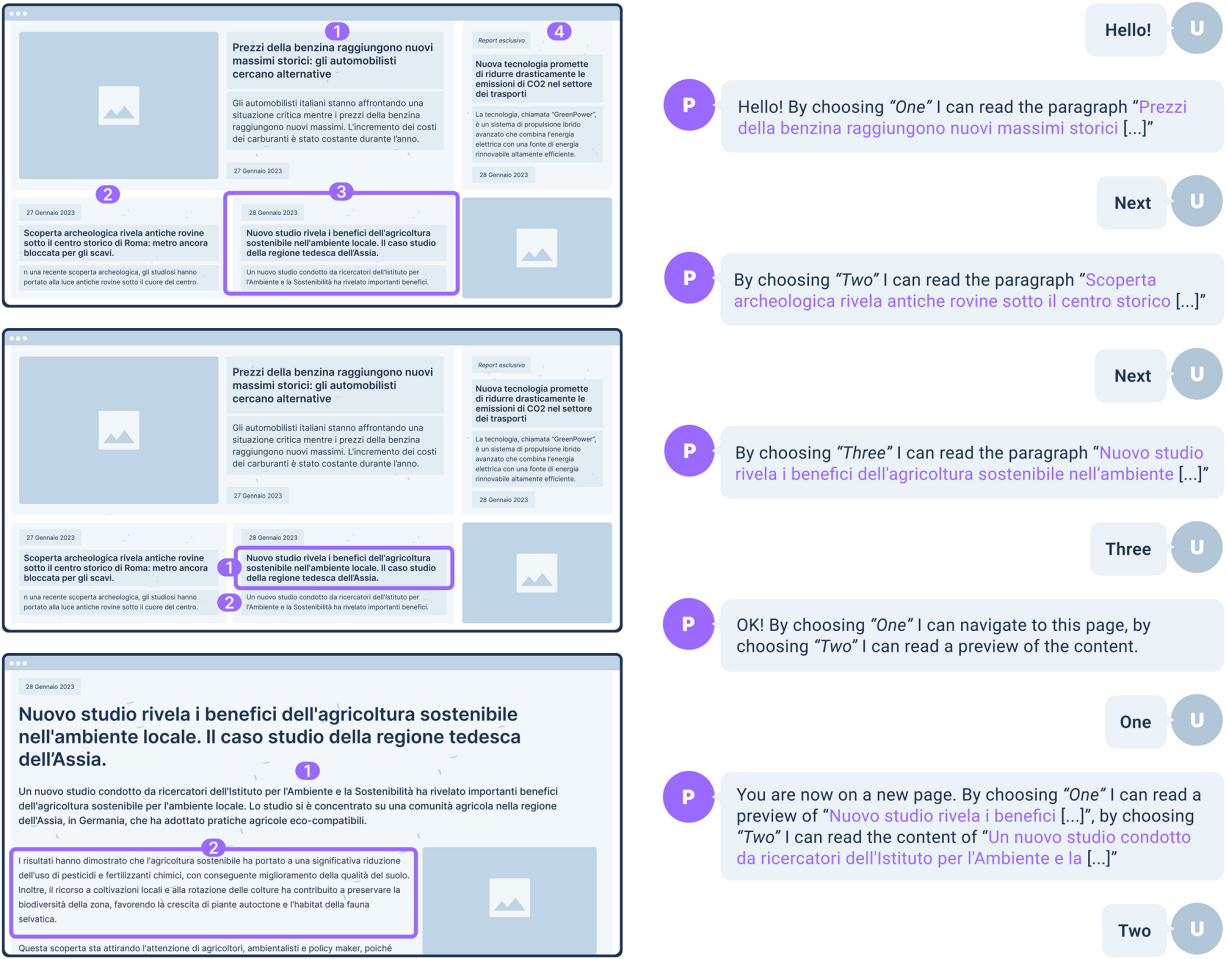
An example of dialogue (translated for clarity from Italian to English) between the user (U) and the *ConWeb* platform (P), for browsing a sample news website. The user moves within the content by pronouncing “Next”, selects a section by pronouncing the corresponding number, and then chooses to navigate to a new web page. The dialog is offered through a computer-generated voice. Legend: It represents an example of dialogue between the user and the ConWeb conversational agent. On the left, there are three Web pages with the visual augmentation mechanisms in action. On the right, the reader can find the corresponding dialogue that guides the interaction with the web pages.

**Figure 3:**
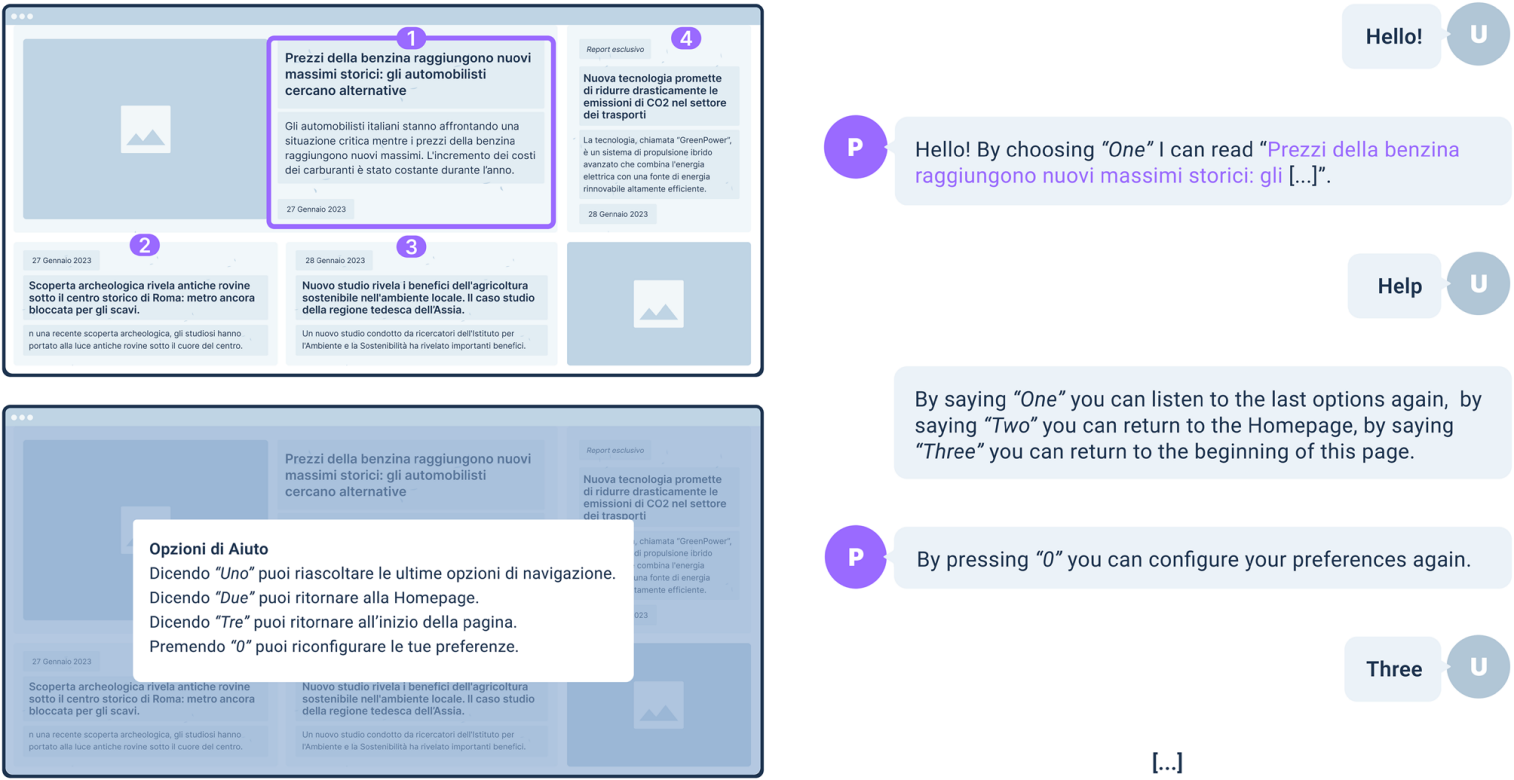
An example of dialogue between the user (U) and the *ConWeb* platform (P). By uttering the word “Aiuto” (“Help” in English), the user accesses the support section. For clarity, the original Italian dialogue is translated into English. It represents an example of dialogue between the user and the ConWeb conversational agent. On the left, there are two Web pages with the visual augmentation mechanisms in action. On the right, the reader can find the corresponding dialogue that guides the interaction with the web pages.

**Visual Augmentation.** It was important to come up with solutions that could leverage the sight ability (even if limited). As depicted in Figs. 2 and 3, a page augmentation mechanism superimposes labels on page visual elements that can be selected. The available options can be *shown visually* and *uttered* by the CA. The user can select vocally or press on the keyboard the relative numbers. A visual augmentation mechanism also highlights the portion of the web page explored by the CA, helping the user follow the dialogue and consume visually the proposed content. Finally, additional visual content can be generated, for example, the pop-up that visually supports the selection of help options shown in Fig. 3.

**Modality configuration.** Given the variability of needs, in an *onboarding process*, the user can choose among three modalities for navigating the Web:

- ***Non-visual Presentation***: the interaction is intended as voice-first, replicating the original *ConWeb* and rendering page content and functionality through voice.
- ***Mixed Presentation***: it provides coordination between vocal and visual interaction; it renders content by voice and augments visually the page to take advantage of the users’ visual abilities and let them localize on the page the locus of action in a given interaction state.
- ***Pure-visual Presentation:*** content is presented only through visual augmentation, without using the voice to list the available options and read the page content aloud. The result is a one-sided conversation (only the user speaks). This modality is intended for people with limited motor yet good visual capabilities, enabling them to browse web pages by voice commands, while leveraging the visual channel for content fruition.

### Multimodal patterns in action

Given the previous patterns, when landing on a website, users can select an interaction modality through a configuration panel; at any time during the navigation, they can change the modality by pronouncing or typing the key “0”.

Figure 2 depicts an interaction example based on the *“Limited-Vocabulary Conversation”* combined with the *“Mixed Presentation”*. When the user reaches a web page, *ConWeb* reads aloud the available elements that can be selected for further exploration and visually highlights them with borders and labels. Either pronouncing the labels or typing the corresponding keys allows the user to select the highlighted elements. For example, in the web page depicted in the upper part of Fig. 2, after scrolling through the initial paragraphs with the command “next”, the user chooses to explore the paragraph labeled with the number “3”. *ConWeb* now enters a new state and outlines the available options for this paragraph (page in the middle). The user select “1” to navigate to a new page reporting the full article and *ConWeb* automatically performs the navigation; in the new page it then segments the content and labels the derived page portions to enable new selections.

Figure 3 then depicts how the user can ask for help by pronouncing the word “Help”. *ConWeb* opens a pop-up window with four options: the first three concern navigation shortcuts. The last one, triggerable by the key “0”, allows the users to configure their preferences again.

## Implementation

To validate the identified multimodal patterns, we developed a prototype that extended the logic for conversational Web browsing already implemented in *ConWeb*. According to this logic, the provision of conversations is based on a page model, the *Conversation-oriented Navigation Tree* (CNT)^8^, that indexes the content segments and the navigational structures available in a given web page and augments them with annotations that help build the dialog system supporting web browsing. In particular, for each accessed web page, *ConWeb* builds a CNT representing the hierarchical nesting of the different page elements, the *conversation nodes*. Each node specifies attributes and descriptions extracted from the web page, which can help render the node content through conversation. This page model showed to be still valid for the new multimodal paradigm; however, the new patterns required extensions that we illustrate in the following.

### Server-side web augmentation

Web page augmentation is enabled by a set of automatically generated annotations that enrich the page HTML and are used within the browser to pre-process the page and build the CNT. *ConWeb* server-side components process the HTML code of the requested page, and in particular its Document Object Model (DOM), and extend it with the annotations. For example, for each tag <p> denoting textual paragraphs, the *ConWeb* adds the following annotations:

- description: it includes the content that the CA presents when the navigation status reaches that element (e.g.: “I can tell you about” plus a summary of the content of the element it refers to).
- keys: it contains the keywords associated with that element. These are representative words extracted from the element content, which are used to match the entities extracted from the user request in the case of natural-language conversation.

Each element with these attributes will correspond to a node in the CNT built on the client side. It is worth noting that if the interaction mode selected by the user requires visual augmentation, the automatic annotation introduces additional elements needed to change the visual appearance of the page, e.g., adding borders for delimiting menus or paragraphs—which is why the process is performed server-side on a copy of the page.

### Client-side web augmentation

The client-side component of the extended *ConWeb* is in charge of building the CNT. It then manages and serves the content presentation based on the browsing modality chosen by the user. The customization of the content presentation is enabled by *policies* that refer to notable browsing states:

**Table 4:** Navigation policy’s features.

Feature	Description
Explicit navigation	Navigation options are no longer listed automatically but the user can request the next one through voice commands (“next”) or typing on the keyboards (arrow key)
Highlighting	The page elements presented by the CA are also visually highlighted
Scrolling	Enables the automatic visual scroll of the screen and centers the page on the content the CA refers to
Labeling	Generates the keywords to be spoken for moving to or selecting a page element and displays labels in association with the specific content
Speed rate	Enables the user to control the CA speech rate.
Silent navigation	Voice commands are enabled but the content is rendered only through the visual paradigm—the CA is silent

The table reports the name of each navigation policy’s feature (on the left) and its description (on the right).

- ***Navigation Policy***. A Navigation Policy is applied every time the user reaches a new page or page element. Starting from a default behavior, that is to present the possible navigation options automatically and only by voice, various features (described in Table 4) can be added depending on the active presentation modality.
- ***Help Policy***. It is applied when the user requests help. The possible options are then presented through the *helper popup*, that shows (visually) and provides an audio description (vocally) of a visual pop-up with all the available help options (see Fig. 3), or the *silent help*, where the help options are only visually presented.
- ***Speech Policy***. It governs the way the users’ speech requests are translated into text. Depending on the users’ configuration for speech-to-text conversions, the framework can use CapisciAMe service or other ASR technologies. In our current prototype, we use Google’s Speech Recognition/Synthesis APIs. The resulting textual request is then sent to the server for its interpretation and the automatic triggering of related browsing actions. It is worth noting that the choice of the ASR modality also influences the way the CNT is built: if *capisciAMe* is selected, only numbers from 1 to 10 can be interpreted for page element selection; therefore, the node representing those elements at each level of the CNT have to be a maximum of 10.

With different combinations of these features, it is possible to define policies that vary on a spectrum from a conversation-only policy to a visual-only policy, thus addressing the varying needs that emerged from the formative studies.

## User-based validation

### Pattern validation

Thanks to the availability of the *ConWeb* extension, in January 2023 we conducted a preliminary evaluation of the identified patterns. We tested the prototype with the 5 participants who had previously participated in our user research activities and therefore knew the ideas behind the new paradigm. They were asked to use *ConWeb* to browse their association website. The sessions were organized remotely.

Each participant installed the *ConWeb* client-side component as an extension of their web browser. Thus, the participants were individually asked to connect to the website URL and browse the website through *ConWeb*. After a brief introduction about the goal of the session, they were provided with general instructions about how to run the browser extension to navigate the website. Then they were assigned *5 browsing tasks*, purposely defined to trigger the adoption of the defined patterns.

#### Task design

In defining the browsing tasks, the researchers first identified a main *browsing scenario* requiring participants to look for information on their association Website. Then, they designed the tasks considering the following steps and criteria :

- *Task Scope and Definition.* First, we identified the results we wanted to achieve and the specific activities that would allow us to gather relevant data. This step laid the foundation for clearly understanding the tasks’ purpose.
- *Task Resources*. The feasibility of the identified activities was analyzed, together with the technical setup needed, considering that the users would have acted remotely.
- *Expected Interaction with ConWeb.* This alignment was crucial to ensure that the identified tasks directly contributed to the intended validation objectives, and involved envisioning how the task would leverage the ConWeb functionality.
- *Expected Navigational Paths.* As for the Interaction with ConWeb, the researchers hypothesized possible navigational paths that the participants would employ to complete the task.
- *Success criteria.* We identified different situations for each task to be considered successful.

An accurate description of the activities is available at https://tinyurl.com/Dysarthria-UserTasks. The participants were thus asked to perform the tasks. Except for **Task a**, designed to let the user get confident with the system, the other 4 tasks were randomly assigned to counterbalance the study and prevent a possible selection bias. The browsing paradigm fully integrated the new features and modality depicted in “Patterns for multimodal web browsing”. The researchers took note of the strategies adopted by the users. A video demonstration of the scenario is available at https://tinyurl.com/Multimodal-DemoScenario

#### Overall user performance and experience

The participants successfully performed the tasks, except for **P1** who reported difficulties while performing Task 1, 2, 3, and 4. The main obstacle was a poor network connection, which created lags between the ongoing and the incoming client-server requests. This situation made the participant feel insecure about the chosen action, he wanted to know what was happening and why the system took so long to respond (**P1**: *“Why is it taking so long? Did I get something wrong?”*. When performing this task, **P2** then asked: *“How do I know that it is loading new information?”* ). The importance of providing both auditory and visual feedback on the system’s state was indeed emphasized by all the participants. **P2** stated: *“Since I gave a command verbally, I’d like to receive auditory feedback, similarly to when a page is loaded after I navigate a link on the visual page”*.

#### Configuring the conversation with the CA

One of the first tasks was choosing the conversation configuration The goal was to test (i) whether the initial setup was easy to understand for the participants, and (ii) whether the choice of the keyboard keys necessary to configure the browser extension was feasible for the participants. All the participants successfully configured the modality, except for **P1**, who needed support because of his highly reduced motor skills (the participant could not type on the keyboard). All of them chose the *conversation based on a limited number of words*, except for **P4**, who went for the *Natural-Language conversation*. Both modalities enabled the participants to browse the web pages successfully. Due to the participants’ diverse pronunciation and speed of the voice, some of the requests were not understood by the CA the first time. Still, after some repetitions, every one of them could accomplish the various designed tasks.

#### Mixed-interaction

The successive tasks were designed to understand how the users would perceive the mixed-interaction paradigm. The users tried all the commands to explore the available areas. When performing the tasks, participants claimed that the website structure and the navigational context were easy to understand. After an initial “learning phase,” they could freely navigate within a web page and move among different ones. All the participants did not understand how to read a paragraph presented by the CA: the participants had to pronounce “Next” after the title of the paragraph, but this was not sufficiently clear. The steps needed to reach the desired content could be perceived as excessive when the website presents a complex layout or multiple div tags. **P2** said: *“I found the process of reading a paragraph a little repetitive: I had to say “Next” several times, even if it was right under the previous text on the screen.”*.

#### Controlling the browser extension

Another challenge was the activation of the microphone to give commands to the CA: **P1, P2, and P3** encountered problems when pressing the “J” key on the keyboard (**P2**:*“I would prefer using the “space bar” since to me it is easier to press”*. **P2** decided to go for a virtual keyboard, which was supported by the framework and helped him complete the tasks (**P2**:*“I like the possibility to use both physical and digital keyboard, depending on my need”*). The participants easily understood basic commands but pointed out they would have liked the commands to be presented *gradually* instead of listening to the options altogether. Although after a few interactions, participants fully understood the audio capturing mechanism, we observed a tendency to repeat the word or phrase if the agent did not provide an immediate response (**P1**: *“I tried again because I thought he didn’t hear me, maybe having an audio clue about the successful audio capture would be handy!”*).

#### Navigation assistance

All participants found that invoking the available help options by pronouncing the word *“Help”* was effective. **P2** stated: *“It is interesting how I can so easily access an overview of all the help commands!”*.

### Applying the system to more complex websites

During the pattern validation, questions emerged about potential problems in interpreting more complex websites. To address these questions, we performed additional testing sessions on other information-intensive Italian websites, i.e., news platforms, museums, online magazines, and public administration services (see Table 5). The goal was not anymore to delve into the need of people living with dysarthria when using the system, yet to understand whether the framework could correctly interpret and let users interact with a complex information-intensive website. We decided then to involve 4 able-bodied users, in the attempt to perform a technical validation, still involving real users that could generate realistic usage situations.

The participants installed the *ConWeb* client-side component as an extension of their web browser. Thus, individually, they were asked to connect to the website URL and browse the website through the multimodal version of *ConWeb*. After a brief introduction about the goal of the validation, they were provided with general instructions about how to run the browser extension to navigate the website (the extension was configured to serve the *Mixed-interaction* and the *Natural Language conversation* modality in all test instances). We asked them to perform five browsing tasks using the ConWeb browser extension on the websites listed in Table 5. The tasks were purposely defined to check the interpretation of all the website’s components, by adapting to the new websites the tasks adopted for the previous evaluation round. A detailed description of the tasks is available at https://tinyurl.com/yf7jbcvs. In total, **140 tasks** were run on the seven websites. The researchers took note of the technical problems that arose during the sessions.

#### Overall performance

85% of, the tasks were successfully performed. Only the content of one site offering banking services (*Poste Italiane*) could not be completely interpreted and read due to the adopted paragraph structure (see Table 5). Participants were happy to see how the system could interpret and browse even more complex websites successfully. However, the sessions brought to light some limitations. In fact, due to the variability of the HTML code, all websites presented issues related to the interpretation of page elements. In some instances, such issues limited the paradigm’s effectiveness but did not disrupt the navigation completely. Table 5 reports a summary of the issues found.

#### Interactivity

This session brought to light also issues related to the interaction with dynamic page elements, such as carousels and drop-down menus, which require a click or hover interaction to display more content or change the displayed content. While correctly announcing all elements, the ConWeb browser extension could not change the state of an element. Thus, there was a lack of visual feedback.

#### Ambiguities and HTML variability

The web page interpretation and augmentation operated by ConWeb are affected by the structure of the HTML. Pages with limited use of semantic tagging appear to be harder to parse, resulting in navigation trees that are more complex and, thus, longer to explore. On the other hand, the use of semantics does not always guarantee proper parsing. In the case of the Wired magazine website, for example, using a section titled *Wired*, just like the link to the home page, created an overlap between different content blocks and functions.

**Table 5:** Websites analysed during the technical validation. The table highlights the tasks completed and the issues found classified as critical or minor, i.e., whether they compromised the execution of the task or not.

Website	Completed tasks	Critical issues	Minor issues
Poste Italiane https://www.poste.it/	a, d	- Empty <a> tags are captured, but cannot be announced properly - Text sections are not captured	The second level in drop-down menus is captured, but not shown
Wikipedia (IT) https://it.wikipedia.org/	a, b, c, d, e	NA	Paragraphs’ titles are not captured
Today https://www.today.it/	a, b, c, d, e	NA	- Page’s refresh slows down the navigation as the CNT has to be re-generated every time - No clear section and article titling hinder the CNT generation
Open https://www.open.online/	a, b, c, d, e	NA	Page’s refresh slows down the navigation the CNT has to be re-generated every time
Wired https://www.wired.it/	a, c, d	- Ambiguous section titling makes navigating paragraphs difficult, as some links are identically labeled	NA
Android https://www.android.com/	a, b, c, d, e	NA	NA
Ministero della Cultura https://www.beniculturali.it/	a, b, c, d, e	NA	Images used as links could not be interpreted as they lacked an alt-text description
Comune di Milano https://www.comune.milano.it/	a, b, c, e	The navigation menu is not interpreted during the page parsing	Accordion sections cannot be opened by the extension

The table reports the websites tested during the technical validation (first column), the task completed by the participants (second column), the main issues found (third column), and the minor issues (fourth column).

## Discussion

By using a human-centered approach, we gradually shifted our focus from the Web browsing habits and the challenges faced by individuals with dysarthria to design patterns that could be integrated into *ConWeb*. In this section, we further reflect on the knowledge gained during this process and report on possible design implications.

### Including people living with impairments in web browsing experiences

Current literature offers a valuable general framework for designing accessible conversational experiences^43–45^. Still, the focus on web browsing is limited, especially for people living with impairments. In our design approach, we focused on understanding the barriers individuals living with dysarthria encounter during web browsing. We observed people participating in our studies and worked with them to co-create conversational and mixed-interaction experiences accommodating their varying needs. Thanks to the specific attention posed to content browsing, also in comparison with other prominent solutions focusing on inclusivity^18,24^, our proposal addresses yet unsolved challenges in web browsing that the human-centered design process highlighted. We deliberately decided to start our research with a small group of five participants living with dysarthria, to develop a profound understanding of their specific requirements, preferences, and limitations when browsing with voice. This approach tailored research and solutions to their needs effectively. Our intention is not to limit the scope of our research solely to dysarthria but rather to utilize the insights gained from this initial phase as a foundation for extending our findings to other types of impairments. Starting with a focused group lays the groundwork for broader research that encompasses various impairments, such as auditory, physical, and cognitive, besides visual disabilities already addressed by our previous research. This iterative and inclusive approach enables us to create AI models and Web Browsing systems that are adaptable, versatile, and responsive to the needs of a diverse range of individuals with impairments.

The preliminary validation suggests that the *Limited-Vocabulary Conversation* and the *Mixed-Interaction* patterns can address prominent challenges faced by users with dysarthria. Participants perceived that the conversational paradigm, possibly combined with the visual augmentation, eases the interaction with the browser and enables them to express their web browsing requests. While these results are preliminary, the response was positive and provided a foundation for larger-scale studies on the impact of the conversational and mixed-interaction paradigm in web browsing scenarios.

### Customizing the conversational paradigm

Going beyond the design of the conversation flow, the presentation and consumption of web content required tailored solutions. The feedback from our evaluation, as well as prior research on voice-based interactions^46,47^, suggest a need for a wider spectrum of voice-based interaction solutions and high-level of control and configurability over the different nuances of the user experience (e.g., pitch, style, speed, multiple channels), which alone would not fit a single persona. During our studies, it was clear how customization is extremely important even within the same group of users. The validation phase highlighted the need for customization even for basic commands, such as microphone activation and the speed of the CA’s voice. People living with dysarthria have needs that can greatly vary, both in terms of speech impairment and other coexisting motor impairments. This variability in the participants’ preferences for input commands and content presentation lets us think that the configurability of conversational elements is the key to improving the conversational experience for any user, and will therefore be the object of further studies and design activities toward extending the inclusivity of web browsing experiences. Future work could also focus on assessing the compatibility of the *ConWeb* browser extension with external hardware and other existing assistive technologies, such as communication software.

### Dialogue fluency and trust in the CA

During the validation sessions, the researchers noticed that the conversation was not designed to inform the users of the state of their requests adequately. The CA did not notify the user when it was loading new content and the delay between the voice command and the communication of the request state generated uncertainty in the participants (**P3: ***“Did the CA get my request? Should I pronounce the command again?”*; **P2:** *“How can I understand if it [the CA] is loading something new?”*). Also, after listing the navigational commands at the beginning of the interaction, the CA did not present the participants with navigational prompts or hints. During the evaluation, this emerged as an issue, as participants who did not remember exactly how to interact with the CA were totally inhibited. When asked, they claimed: *“I expected the CA to give me instructions. The way it abruptly stopped created confusion”*. Participants expected the CA to keep talking to and guiding them during the various activities. Conversation breaks created a sense of bewilderment, which was palpable also during the subsequent tasks (the participants started asking for confirmation and were in general more insecure about their choices). Adding such prompts would improve the CA discoverability and user confidence in the interaction with the agent. Future investigations could examine whether this trend is confirmed with a larger and diversified user sample.

### Information overload

The CA listed all the commands at the beginning of the interaction, as an intro. This proved to be too much for the users to remember (**P3:** *“It is quite a lot of information to remember”*). Following an incremental approach, the CA could be revised to provide only the basic commands of “hello” (to start the conversation) and “help”, while the rest of the commands could be introduced progressively as the users interact with the various elements (for example, the CA could give instructions of how to read a paragraph only when the user selects a paragraph to read, and not before).

### Recommending and adapting the interaction modality

As said before, speech and motor disorders can vary significantly from user to user. In some cases, a user interacts using natural language without limitations, yet problems might arise when pronouncing a single word. Our validation let us perceive that forcing the participants to navigate using a limited vocabulary might be frustrating. To overcome this type of situation, the limited vocabulary can be triggered based on the observation of the user experience. In the future, mechanisms for the on-the-fly analysis of user interaction could be devised so that the CA can proactively recommend which interaction modality best suits the user behavior. For example, when not understanding a command a predefined number of times, the CA could ask the user if he/she wants to move to the limited-vocabulary mode and the mixed interaction. The flexibility that the policy-based mechanism has introduced in the *ConWeb* could thus be used to limit the users’ frustration of not being understood, besides improving the overall sense of inclusion.

#### Limitations

Even if the gained insights are encouraging, the conducted evaluation presents limitations. Firstly, the evaluation was conducted only on one website featuring a very consistent and regular structure and providing mainly textual content. This might not be fully representative of the broad range of content available on the Web. Although the last validation session showed that many websites are already, at least partially, explorable with the defined patterns, the current prototype does not address a few page elements like forms, thus limiting its scope of usage to informative websites, such as news pages or blogs. The Web, instead, presents a wide variety of content formats on which future investigations will be performed to make them also accessible through voice interaction. Lastly, this was a small-scale user-centered research that focused on a limited sample of users, to deeply understand their needs. The number of participants is similar to that of other studies found within the literature. However, it is, indeed, limited and all the participants self-declared as male. Further large-scale evaluation, involving both a greater and more representative sample of users and focusing on different websites, will allow us to assess the validity of the paradigm further.

## Conclusions

This paper illustrates the design of multimodal intelligent interfaces for web browsing addressing the needs of people with dysarthria. The research followed a human-centered process that involved individuals living with dysarthria in the definition of the new Web browsing patterns and emphasized the importance of collaborating with them to create effective solutions, starting with a limited group of participants to deeply understand the needs and specificities of people living with dysarthria. In comparison with other proposed approaches, like the Grid 3 communication tool, this research aims to leverage information already present on the Web and make it accessible through augmentation and generative techniques, even in the absence of ad-hoc tagging of the HTML code. Our overarching goal is to push forward the idea of inclusive web browsing, thanks to intelligent technologies that can natively offer web augmentation.

Currently, our efforts are focused on consolidating the prototype of the *ConWeb* by addressing the existing limitations that emerged from the conducted studies. Further studies, with a higher number and a more diverse group of participants, will focus on validating and consolidating the findings achieved so far, and exploring how nuances in user needs can be addressed through policy-based, flexible browsing environments.

We are confident that the flexibility devised for our platform can help offer “open” interaction paradigms on the Web, which can be customized to address the varying needs deriving from different forms of disabilities. Our ultimate goal is, therefore, to investigate how conversational AI and web augmentation can effectively extend the browsing possibilities for people living with impairments and make the Web truly inclusive. While current AI developments have undoubtedly focused on creating powerful and sophisticated models, there is a growing concern that they often overlook the specific needs and challenges faced by individuals, especially the ones living with impairments. The emphasis on large-scale AI research and deployment tends to prioritize generalization and performance across a wide range of tasks. Still, this approach can inadvertently neglect the unique requirements and accessibility considerations of people living with disabilities. It is crucial to recognize the significance of conducting research on a smaller scale to address the gaps and needs of this particular category. By delving into the specific challenges faced by individuals with impairments, AI researchers and developers can create more inclusive and equitable technologies. This approach involves understanding the diverse range of impairments, whether visual, auditory, physical, or cognitive, and developing AI systems tailored to accommodate these specific requirements.

By focusing on small-scale and user-centered research, AI can be harnessed to assist people with disabilities in their daily lives. This study wants to foster a methodological and technological framework where intelligent technologies are truly inclusive and empower individuals to participate fully in society, access information, and contribute with their valuable insights and talents.

## Data Availability

Davide Mulfari is a Ph.D. student enrolled in the program on Health and life sciences at the Università Campus Bio-Medico di Roma, XXXVII cycle, within the Italian Ph.D. in Artificial Intelligence. The datasets generated and/or analysed during the current study are not publicly available due to the sensitive nature of the topic and preferences of participants but are available from the corresponding author on reasonable request.
